# Retraction: Synthesis and characterization of titanium dioxide nanoparticles from *Bacillus subtilis* MTCC 8322 and its application for the removal of methylene blue and orange G dyes under UV light and visible light

**DOI:** 10.3389/fbioe.2026.1858425

**Published:** 2026-04-30

**Authors:** Frontiers Editorial Office

The Journal retracts the 2024 article cited above.

Following publication, concerns were raised regarding the scientific validity of the article. An investigation was conducted in accordance with Frontiers’ policies.

It was found that the complaints were valid and that the article does not meet the standards of editorial and scientific soundness for Frontiers in Bioengineering and Biotechnology; therefore, the article has been retracted.

This retraction was approved by the Chief Editors of Frontiers in Bioengineering and Biotechnology and the Chief Executive Editor of Frontiers. The authors do not agree to this retraction.

